# Antioxidant and anti‐inflammatory peptide fraction from oyster soft tissue by enzymatic hydrolysis

**DOI:** 10.1002/fsn3.1710

**Published:** 2020-06-13

**Authors:** Bingjun Qian, Xin Zhao, Ye Yang, Chongchong Tian

**Affiliations:** ^1^ Institute of Biomedical Technology Jiangsu Vocational College of Medicine Yancheng China; ^2^ Department of Food Science and Engineering Shanghai Jiao Tong University Shanghai China

**Keywords:** amino acid composition, anti‐inflammation ability, antioxidant activities, bioactive peptide fractions, oyster soft tissue hydrolysates

## Abstract

Recent studies have confirmed that the peptide fractions derived from marine organisms exhibit good antioxidant and anti‐inflammatory activity, and oyster is an excellent nutrient resource with high‐protein content. In this study, the peptide fractions from oyster soft tissue were prepared after hydrolysis by pepsin (pH 2, 37°C), trypsin (pH 8, 37°C), and Maxipro PSP (pH 4.2, 50°C) with the optimized parameters (enzyme‐to‐substrate (E/S) ratio, 1:100 (w/w); hydrolysis time, 4 hr), respectively. Four fractions named as PEP‐1, PEP‐2, TRYP‐2, and MIX‐2 were obtained after separation with elution consisting of 20% or 40% ethanol. The MIX‐2 exhibited the highest hydrophobicity correlated well with its hydrophobic amino acid content, and TRYP‐2 exhibited much better antioxidant activity than other three elution samples. Furthermore, all of the bioactive peptide fractions were noncytotoxic and could selectively repress pro‐inflammatory mediators, TNF‐α, IL‐1β, IL‐6, and i‐NOS, at transcription level in RAW264.7 macrophage cells after LPS stimulation. The result suggests that the peptide fraction TRYP‐2 from oyster soft tissue hydrolysates might be a potential resource for natural anti‐inflammatory components.

## Graphical abstract

In this study, the peptide fraction TRYP‐2 we obtained from oyster protein hydrolysates might be a potential resource for natural anti‐inflammatory components.

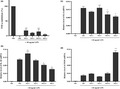

## 1 INTRODUCTION

Free radical reactions can cause much adverse effect to organisms by destroying the biological molecules, and free radicals play vital roles under normal physiological conditions. With aging or by other adverse factors stimulation, the free radicals are excessively produced in vivo and the redox homeostasis is destructed, which might lead to various cellular components suffering from oxidative damage (Chai, Law, Wong, & Kim, 2017; Giordano et al., 2018). Oxidative damage is considered to be a major cause of inflammatory events implicated in a large number of diseases, such as cancer, diabetes, neurological malfunction, hypoimmunity, and other aging‐associated diseases (Luca, Luca, & Calandra, 2015; Weidinger & Kozlov, 2015; Ye, Zhang, Townsend, & Tew, 2015). Free radical scavengers, also named as preventive antioxidants, act as a vital role in preventing those diseases caused by oxidative damage (Bo et al., 2016). Natural antioxidants are increasingly favored over synthetic ones for health food industries (Agyei, Danquah, Sarethy, & Pan, 2015; Zhong & Shahidi, 2015).

In recent years, increasing antioxidant peptides or peptides fractions have been explored from numerous oceanic fishes and invertebrates (Asha et al., 2016; Halim, Yusof, & Sarbon, 2016; Park, Kim, Ahn, & Je, 2016). The bioactive peptides exhibit various biological activities, mainly including antioxidative, antihypertensive, anticancer, antidiabetic, and antimicrobial properties (Agrawal, Acharya, Adholeya, Barrow, & Deshmukh, 2017; Agrawal, Adholeya, & Deshmukh, 2016; Blunt, Copp, Keyzers, Munro, & Prinsep, 2017; Gogineni & Hamann, 2018).So, aside from the plant polyphenols, the natural proteins, derived from plants or animals, are the other antioxidants resources. And, enzymatic hydrolysis is a simple and effective process for large‐scale production of bioactive peptides from marine proteins (Kim & Wijesekara, 2013).

Oysters are rich in vitamins, minerals, and protein, but less fat. It is an important source of excellent quality nutrients in many areas (Cheong, Xia, & Liu, 2017; Qian, Li, Liu, & Xu, 2016; Wang et al., 2014). In the present study, we aimed to obtain the hydrophobic peptides factions from oyster proteins hydrolysates by enzyme hydrolysis and various concentrations of ethanol elution after DA201‐C macroporous absorption. The antioxidant activity and anti‐inflammatory effects of the bioactive peptides fractions were respectively evaluated by DPPH scavenging ability test, FRAP and ORAC assay and through inflammation‐associated genes (TNF‐α, IL‐1β, IL‐6, and i‐NOS) expression analysis in RAW 264.7 macrophage cells.

## 2 MATERIALS AND METHODS

### 2.1 Materials

Dried oysters soft tissues (*Crassostrea talienwhanensis*) were provided by Hainan Coconut Island Brewing Co., Ltd. The reagents sodium dodecyl sulfate (SDS), HCl, NaOH, NaCl, ethanol, methanol, potassium ferricyanide, ferric chloride, and fluorescein were purchased from Sinopharm Chemical Reagent Co., Ltd. Phosphoric acid, copper sulfate, potassium sulfate, and disodium hydrogen phosphate were purchased from Shanghai Ling Feng Chemical Reagent Co., Ltd. Pepsin (EC 3.4.23.1, 1:15,000 from Porcine Stomach) and Trypsin (EC 3.4.4.4, 1:2,500 from porcine pancreas) were purchased from Shanghai SenGon Bioengineering Co., Ltd. Maxipro (EC 3.4.21.xx), a proline‐specific endoproteinase from* Aspergillus niger* (PSP), was obtained from DSM. DA201‐C was kindly provided by Jiangsu Suqing Water Treatment Engineering Co., Ltd. 1,1‐Diphenyl‐2‐picrylhydrazyl free radical (DPPH) was purchased from A. Johnson Matthey Company. Trolox, ascorbic acid, lipopolysaccharide (LPS), penicillin, and streptomycin were acquired from Sigma Chemical Co.. Dulbecco's modified eagle's medium (DMEM), fetal bovine serum (FBS), trypsin–EDTA, and glutamine were obtained from GIBCO (Life Technologies). Cell Counting Kit‐8 (CCK‐8) was purchased from Biyuntian Biotech Co., Ltd. Mouse TNF‐α ELISA kit was purchased from Affymetrix eBioscience. All reagents used were of analytical grade.

### 2.2 Preparation and isolation of the bioactive peptide fractions from oyster protein

The dried oyster soft tissues were ground with a grinder and sifted with a 80‐mesh sieve. The obtained powders contained 46.87 ± 2.65% (w/w) protein (Qian et al., 2016). Twelve‐gram dried oyster soft tissue powders were dissolved in 100 ml of phosphate buffer solution (PBS) and then hydrolyzed with pepsin (pH 2, 37°C), trypsin (pH 8, 37°C), and Maxipro (pH 4.2, 50°C). The enzyme‐to‐substrate (E/S) ratio and hydrolysis time optimized as 1:100 (w/w) and 4 hr using degree of hydrolysis (DH) as indicator, respectively, for these enzymes. The enzymatic hydrolysis reaction was terminated by incubation in boiled water and then cooled immediately. After a centrifugation of the obtained hydrolysates (18,000 *g*; 10 min), the supernatant layer was filtered using filter cloth and stored at −20°C until further analysis. After preparation, the hydrolysates were absorbed by macroporous adsorption resin DA201‐C. To obtain the hydrophobic peptides fractions, the prepared resin was eluted by 20% ethanol firstly until the OD_280_ was less than 20. Subsequently, the single peak fraction detected at 220 nm was collected and merged together by an elution with 40% and 60% ethanol in turn. The peptide fractions were vacuum freeze‐dried into powder and stored at 4°C before they were weighted and dissolved in PBS (pH 7.4) for further analysis.

### 2.3 Amino acid composition and the degree of hydrophobicity in the bioactive peptide fractions

The peptides fractions were further hydrolyzed by 6 M HCl at 110 ± 1°C for 22 hr, and the hydrolysates were used for the amino acid composition analysis with a L‐8900 automatic amino acid analyzer (Hitachi), and the Ney's *Q* value is calculated for evaluating the degree of hydrophobicity (Ney, 1971).

### 2.4 DPPH radical (DPPH) scavenging capacity assay

The scavenging effect of the bioactive peptide fractions on 2,2‐diphenyl‐1‐picrylhydrazyl radical (DPPH) was determined according to our laboratory protocols described previously (Qian, Pan, Cai, & Jing, 2017). In brief, 1 ml of 1 mg/ml each sample was mixed well with 4 ml of 0.076 mM DPPH dissolved in methanol and incubated for 30 min at room temperature. The absorbance of the resulting solutions was measured at 516 nm against methanol as blank. The standard curve was constructed using a series of concentrations of ascorbic acid to replace the tested samples, with which ascorbic acid equivalents (AAE) were calculated. The results were expressed as μg AAE/mg of the bioactive peptide fractions. Three replicates were finished for each test.

### 2.5 The Ferric Reducing Antioxidant Power (FRAP) assay

The FRAP assay was performed in accordance with the laboratory protocol represented previously with slight modify. The 0.5 ml of 1 mg/ml each sample was added to equivoluminal phosphate buffer (pH = 6.6) and 0.1% potassium ferricyanide solution and mixed well, followed by an incubation at 50°C for 20 min. The reaction mixture was then cooled as quickly as possible in an ice bath. 0.5 ml trichloroacetic acid solution was added to the reaction mixture and mixed well. Subsequently, 0.8 ml ferric chloride solution and 4 ml deionized water were added successively, mixed well and let stand for 10 min. The absorbances of the resulting solutions were measured at 700 nm. The standard curve was constructed using a series of concentrations of ascorbic acid to replace the tested samples, with which ascorbic acid equivalents (AAE) were calculated. The results were expressed as μg AAE/mg of the bioactive peptide fractions. Three replicates were finished for each test.

### 2.6 Oxygen Radical Absorbance Capacity (ORAC) Assay

The ORAC values were conducted by using fluorescein as the fluorescent probe according to a laboratory protocol described previously (Moore et al., 2005). The peptides fractions and Trolox were respectively dissolved in deionized water to a concentration of 1 mg/ml and a series of concentrations, and fluorescein and 2,2′‐azobis(2‐amidinopropane) hydrochloride (AAPH) were dissolved with 75 mM phosphate buffer (pH 7.4). In the final 200 μl of assay system, there were 4 nM fluorescein, 153 mM AAPH, 25 μl of 1 mg/ml sample, or deionized water for reagent used as blank. After reaction, the fluorescence of the final reactants for 2 hr with 1‐min interval. Excitation and emission wavelengths were respectively set as 485 and 520 nm. ORAC values were reported as μmol of Trolox equivalents (TE) per mg of peptides factions. Three replicates were finished for each test.

### 2.7 Cell culture and cell viability assay

Macrophage cell line RAW264.7 provided by Fudan University Shanghai Cancer Center (Shanghai, China) was cultured in DMEM containing 10% FBS, 2 mM glutamine, 100 U/ml penicillin, and 100 μg/ml streptomycin. The incubation parameters were set as temperature 37°C, relative humidity 95%, and CO_2_ 5%.

The cell viability was determined by CCK‐8 according to the supplier's instructions. In brief, RAW264.7 macrophage cells were seeded in 96‐well plate at a density of 1 × 10^4^ cells/well. After incubation for 24 hr, the cells were exposed to the different concentrations of bioactive peptide fractions (1 mg/ml, 0.6 mg/ml and 0.2 mg/ml) or vehicle for another 24 hr‐incubation. Then, 10 μl of CCK‐8 solution was added to each well followed by a 4 hr‐incubation at 37°C. The absorbance was read at 450 nm. Each group was set up for three repetitions.

### 2.8 Determination of TNF‐α production

RAW 264.7 macrophage cells were seeded in 24‐well plates at no less than 1 × 10^5^ cells/ml and pretreated with 0.6 mg/ml peptide fraction, or equal volume of PBS as control for 30 min. Subsequently, 10 ng/ml LPS was added to stimulate cells for 6 hr. The cell supernatant was collected and centrifuged at 10,000 rpm for 10 min. The obtained cell supernatant was used to assess the secretion of TNF‐α with mouse TNF‐α ELISA kit according to the manufacturer's protocol (eBioscience, USA). The percentage inhibition of TNF‐α secretion was calculated using the following formula:

$$\text{Inhibition}\;\left(\%\right)=\frac{\text{TNF}-\alpha_{\text{control}}-\text{TNF}-\alpha_{\text{sample}}}{\text{TNF}-\alpha_{\text{control}}}\times 100$$

### 2.9 Real‐time PCR analysis for pro‐inflammatory gene expression

RAW264.7 macrophage cells were treated as described in the “Determination of TNF‐α production” section. The treated cells were harvested for RNA isolation using RNeasy Mini Kit (Qiagene). Advanced cDNA Synthesis kit (Takara) was used to reverse transcribe complementary DNA. Real‐time PCR analysis of pro‐inflammatory cytokines, interleukin 1β (IL‐1β), interleukin 6 (IL‐6), and inducible Nitric Oxide Synthase (i‐NOS), mRNA expression level was performed using SYBR Green qRT‐PCR mix (Bio‐Rad) according to previous report (Zhang et al., 2012). The primer sequences were as follows: GADPH (Forward: 5′‐CCATGGAGAAGGCTGGG‐3′, Reverse: 5′‐CAAAGTTGTCATGGATGACC‐3′); IL‐1β (Forward: 5′‐GGCGATACCTCAGCAACCG‐3, Reverse: 5′‐CTAAGGCGAAAGCCCTCAAT‐3′); IL‐6 (Forward: 5′‐CACGGCCTTCCCTACTTCAC‐3′, Reverse: 5′‐TGCAAGTGCATCATCGTTGT‐3′); i‐NOS (Forward: 5′‐ AGCTCCTCCCAGGACCACAC‐3, Reverse: 5′‐ACGCTGAGTACCTCATTGGC‐3′). Experiment was repeated three times for each sample. The result was normalized to the reference gene GADPH.

### 2.10 Statistical analysis

Three replicates were finished for each test. Data were expressed as mean ± *SD*, and analysis of variance (AOV) was finished using SPSS 13.0 (SPSS, Chicago, IL, USA). The least significant difference (LSD) test was used to determine the difference between experimental groups. *p* < .05 was considered statistically significant.

## 3 RESULTS

### 3.1 Preparation and fractionation of the bioactive peptide fractions from oyster protein

**FIGURE 1:**
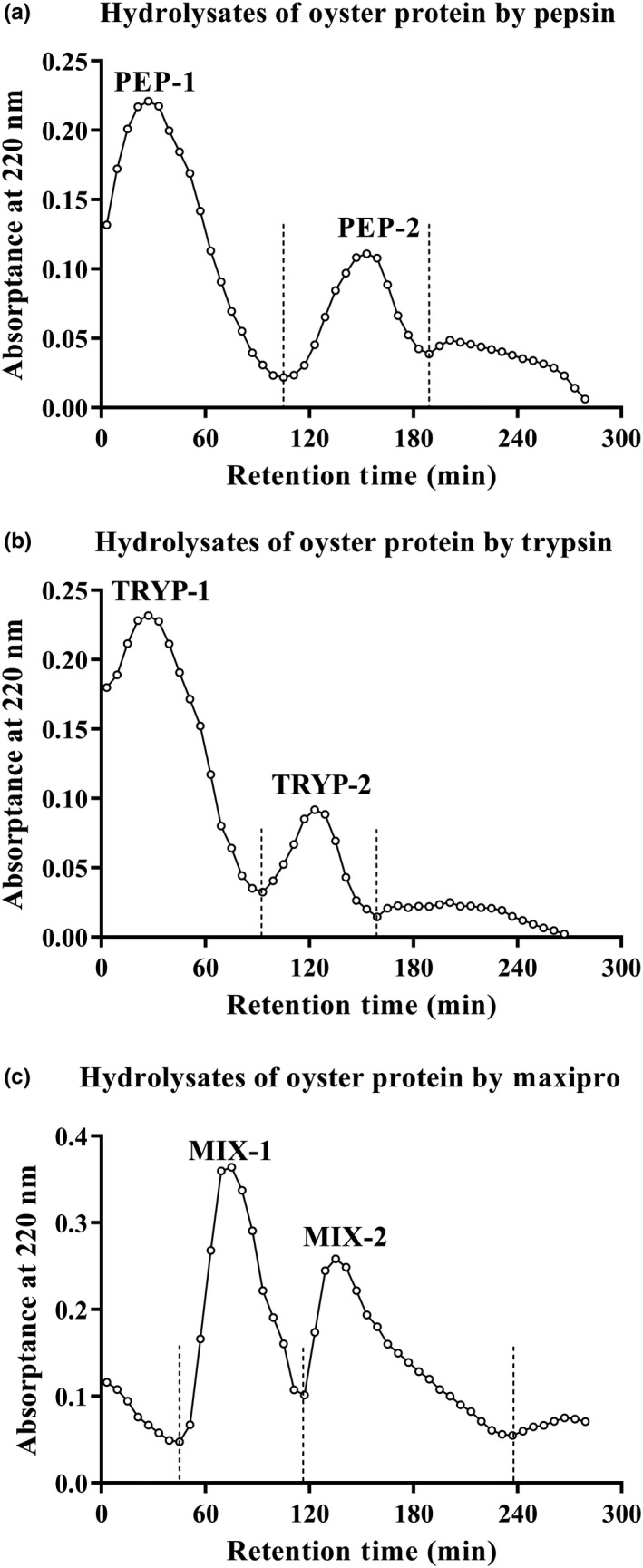
Elution diagram of the oyster protein hydrolysates using macroporous adsorption resin DA201‐C. Bioactive peptide fractions, PEP‐1 and PEP‐2 (a), TRYP‐1 and TRYP‐2 (b), MIX‐1 and MIX‐2 (c); mobile phase, 20%, 40% and 60% ethanol, were respectively used to elute the single peak faction, at a flow rate of 5 ml/3 min; Detection, absorbance at 220 nm; Circle indicates OD_220_

Oyster protein was hydrolyzed with pepsin, trypsin, and Maxipro PSP, and the final hydrolysates were absorbed by macroporous adsorption resin DA201‐C. The different hydrophobic peptides fractions were eluted by 20%, 40%, and 60% ethanol. Absorption peaks of pepsin, trypsin, and Maxipro PSP at 220 nm were collected at the concentration of 20% and 40% ethanol, while no peak was collected at 60% ethanol (Figure 1). The bioactive peptide fractions from pepsin hydrolysates eluted with 20% and 40% ethanol were named as PEP‐1 and PEP‐2, respectively. The bioactive peptides fractions from tryptic hydrolysates eluted with 20% and 40% ethanol were named as TRYP‐1 and TRYP‐2, respectively. The bioactive peptides fractions from Maxipro PSP hydrolysates eluted with 20% and 40% ethanol were named as MIX‐1 and MIX‐2, respectively. After freeze‐drying, it was found that PEP‐1, PEP‐2, TRYP‐2, and MIX‐2 could be frozen to powder state, while TRYP‐1 and MIX‐1 were viscous and difficult to separate. This might be due to the polysaccharides in the ingredients. Therefore, we conducted in‐depth research on PEP‐1, PEP‐2, TRYP‐2, and MIX‐2.

### 3.2 Amino acid composition of the bioactive peptide fractions

The amino acid compositions of PEP‐1, PEP‐2, TRYP‐2, and MIX‐2 are summarized in Table 1. Apart from essential amino acid Trp, the other seven ones were present in PEP‐1, PEP‐2, TRYP‐2, and MIX‐2. In addition, the bioactive peptide fractions contained a small amount of the free amino acid taurine (Tau). The contents of hydrophobic amino acids in PEP‐1, PEP‐2, TRYP‐2, and MIX‐2 were 13.01 g/100 g, 30.98 g/100 g, 31.04 g/100 g, and 33.02 g/100 g, respectively. The grand average of hydrophobicity assessed by Q value was 4.41 kJ/mol, 4.68 kJ/mol, 4.58 kJ/mol, and 4.85 kJ/mol, respectively. What is more, there are also a relatively high amount of negatively charged amino acids, namely Glu and Asp, present in the four peptide fractions, which accounts for 9.95%, 22.77%, 24.59%, and 23.11% of the total amino acids, respectively.

**TABLE 1:** Amino acid composition of the bioactive peptide fractions from oyster

Amino acid	Hydrophobicity value (kJ/mol)	Relative molecular mass (g/mol)	Amino acid content (g/100 g)			
			PEP‐1	PEP‐2	TRYP‐2	MIX‐2
Asp	2.26	133.1	3.88	9.59	10.75	10.03
Thr^b^	1.84	119.1	1.91	3.84	3.79	3.76
Ser	0.17	105.1	2.16	3.89	4.56	3.87
Glu	2.30	147.1	6.07	13.18	13.84	13.08
Gly	0	75.1	2.25	5.64	5.97	5.63
Ala^a^	3.06	89.1	2.41	3.91	3.89	3.49
Cys	4.19	121.2	0.09	0.15	0.19	0.08
Val^a^, ^b^	7.07	117.1	1.88	3.90	4.08	4.05
Met^a^, ^b^	5.44	149.2	0.85	1.59	1.43	1.58
Ile^a^, ^b^	12.43	131.2	1.58	3.44	3.72	3.95
Leu^a^, ^b^	10.13	131.2	2.67	5.74	5.67	6.45
Tyr^a^	12.01	204.2	1.16	3.56	3.33	4.13
Phe^a^, ^b^	11.09	165.2	0.93	3.10	2.81	3.76
Lys^b^	6.28	146.2	4.26	3.03	3.12	2.15
His	2.09	155.2	0.85	1.17	1.35	1.47
Arg	3.06	174.2	3.27	3.15	2.81	2.03
Pro^a^	10.88	115.1	1.53	5.74	6.11	5.61
Tau		125.15	0.19	0.48	0.37	0.21
Total amount of hydrophobic amino acid (g/100 g)			13.01	30.98	31.04	33.02
Grand average of hydrophobicity value *Q* (kJ/mol)			4.41	4.68	4.58	4.85

^a^ Hydrophobic amino acids.

^b^ Essential amino acids.

### 3.3 Antioxidant activity of the peptide fractions PEP‐1, PEP‐2, TRYP‐2, and MIX‐2 in vitro

**FIGURE 2:**
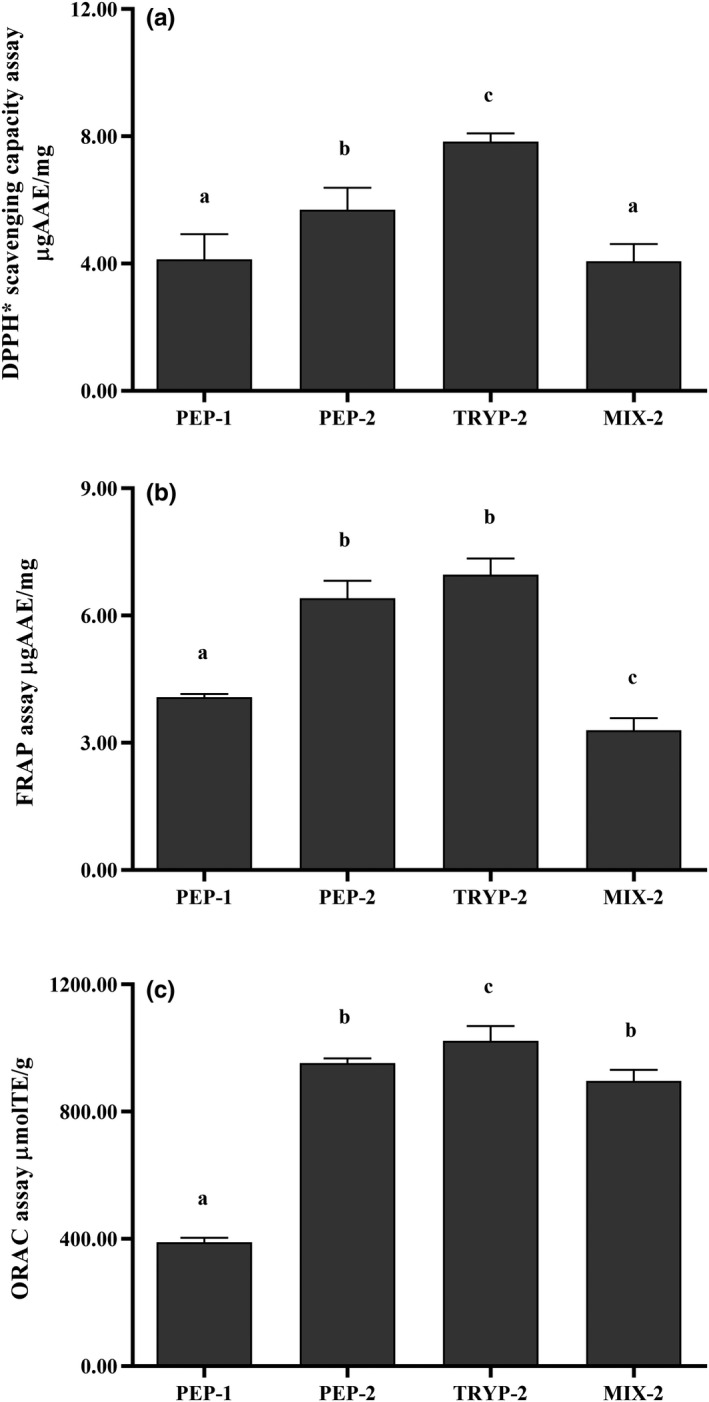
Antioxidant capacity assay of the bioactive peptide fractions (PEP‐1, PEP‐2, TRYP‐2 and MIX‐2). (a) DPPH scavenging capacity assay. (b) Ferric Reducing Antioxidant Power (FRAP) assay. (c) Oxygen radical absorbing capacity assay. Results are expressed as micromoles of Ascorbic acid equivalents (AAE) or Trolox equivalents (TE) per milligram of the bioactive peptides fractions. All tests were conducted in triplicate, and mean values are used. The vertical bars represent the standard deviation of each data point. Values marked by the same letter are not significantly different (*p* > .05)

The free radical scavenging activity of the bioactive peptide fractions was determined by DPPH^•^ scavenging assay. As shown in Figure 2a, all the bioactive peptide fractions showed potent DPPH scavenging activities. The strongest DPPH^•^ scavenging capacity was observed for TRYP‐2 (7.83 ± 0.26 μg AAE/mg) among the 4 peptide fractions, followed by PEP‐2 (5.69 ± 0.69 μg AAE/mg), PEP‐1 (4.13 ± 0.79 μg AAE/mg), and MIX‐2 (4.07 ± 0.54 μg AAE/mg). What is more, the DPPH^•^ scavenging activity was significantly higher for TRYP‐2 compared with that of PEP‐1, PEP‐2, and MIX‐2 (*p* < .05).

FRAP assay was also performed to investigate the reducing capacity of the bioactive peptide fractions. As depicted in Figure 2b, all the bioactive peptide fractions could efficiently reduce Fe^3+^ to Fe^2+^, among which TRYP‐2 exhibited the strongest reducing power with a FRAP value of 6.96 ± 0.38 μg AAE/mg compared with that of PEP‐2 (6.41 ± 0.41 μg AAE/mg), PEP‐1 (4.08 ± 0.07 μg AAE/mg), and MIX‐2 (3.30 ± 0.28 μg AAE/mg), respectively. The FRAP values for TRYP‐2 and PEP‐2 were significantly higher than that of PEP‐1, MIX‐2. The results showed that it was consistent with those of the DPPH• scavenging activity assay in this study.

The ORAC assay was performed to test the scavenging capacity of the bioactive peptide fractions against peroxyl radicals. Commonly, higher the ORAC value of chemicals means that they possess better antioxidant property. TRYP‐2 (1,022.03 ± 47.43 μmol TE/mg) exhibited higher (*p* < .05) ORAC than PEP‐2 (952.44 ± 15.43 μmol TE/mg), MIX‐2 (896.41 ± 35.24 μmol TE/mg), and PEP‐1 (389.81 ± 13.95 μmol TE/mg) (Figure 2c). The ORAC value of the bioactive peptide fractions was in accordance with our previous DPPH radical scavenging and FRAP data.

### 3.4 Cytotoxicity of the peptide fractions PEP‐1, PEP‐2, TRYP‐2, and MIX‐2

**FIGURE 3:**
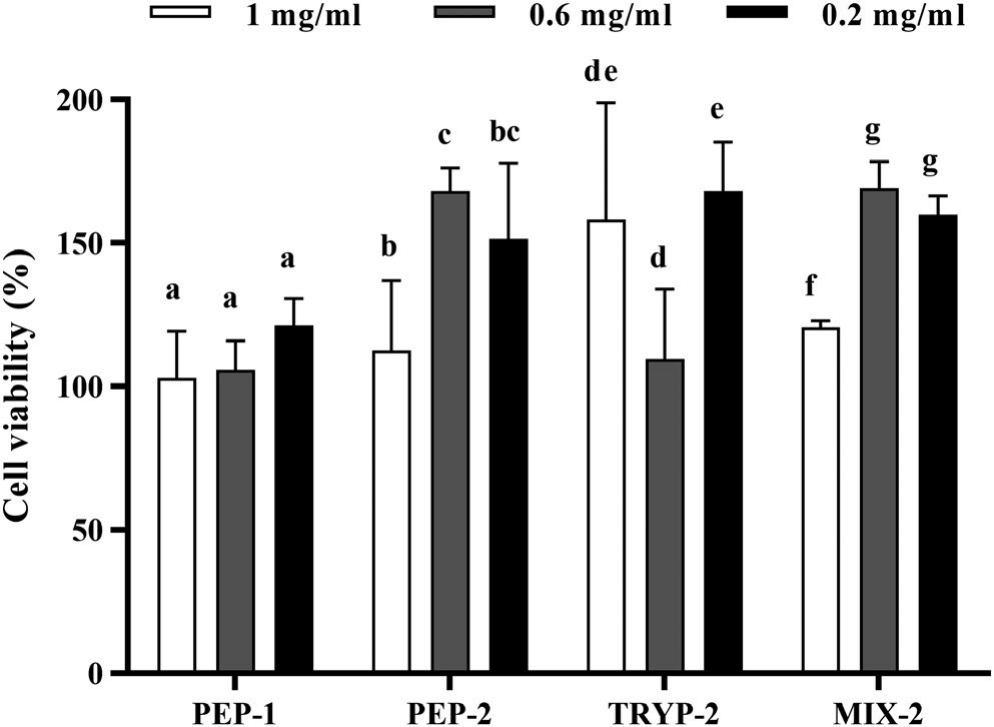
Cytotoxicity of the bioactive peptide fractions in RAW 264.7 mouse macrophage cells. The final concentrations of the bioactive peptide fractions were 1 mg/ml, 0.6 mg/ml, and 0.2 mg/ml in the initial culture media. The absorbance was read at 450 nm. All tests were conducted in triplicate, and mean values are used. The vertical bars represent the standard deviation of each data point. Values marked by the same letter are not significantly different (*p* > .05)

Cytotoxicity of the bioactive peptide fractions was assessed by CCK‐8 assay, and the result indicated that the bioactive peptide fractions at different concentrations (1 mg/ml, 0.6 mg/ml, and 0.2 mg/ml) did not reduce the viability of RAW264.7 macrophage cells (Figure 3). What is more, the OD values were increased after addition of the four peptide fractions at indicated concentrations exhibiting no cytotoxicity. It is noted that the bioactive peptide fractions at 0.6 mg/ml had the most obvious effect on cell proliferation, so we choose the concentration of 0.6 mg/ml for further anti‐inflammatory experiments.

### 3.5 Effects of the bioactive peptide fractions on cytokine genes expression

**FIGURE 4:**
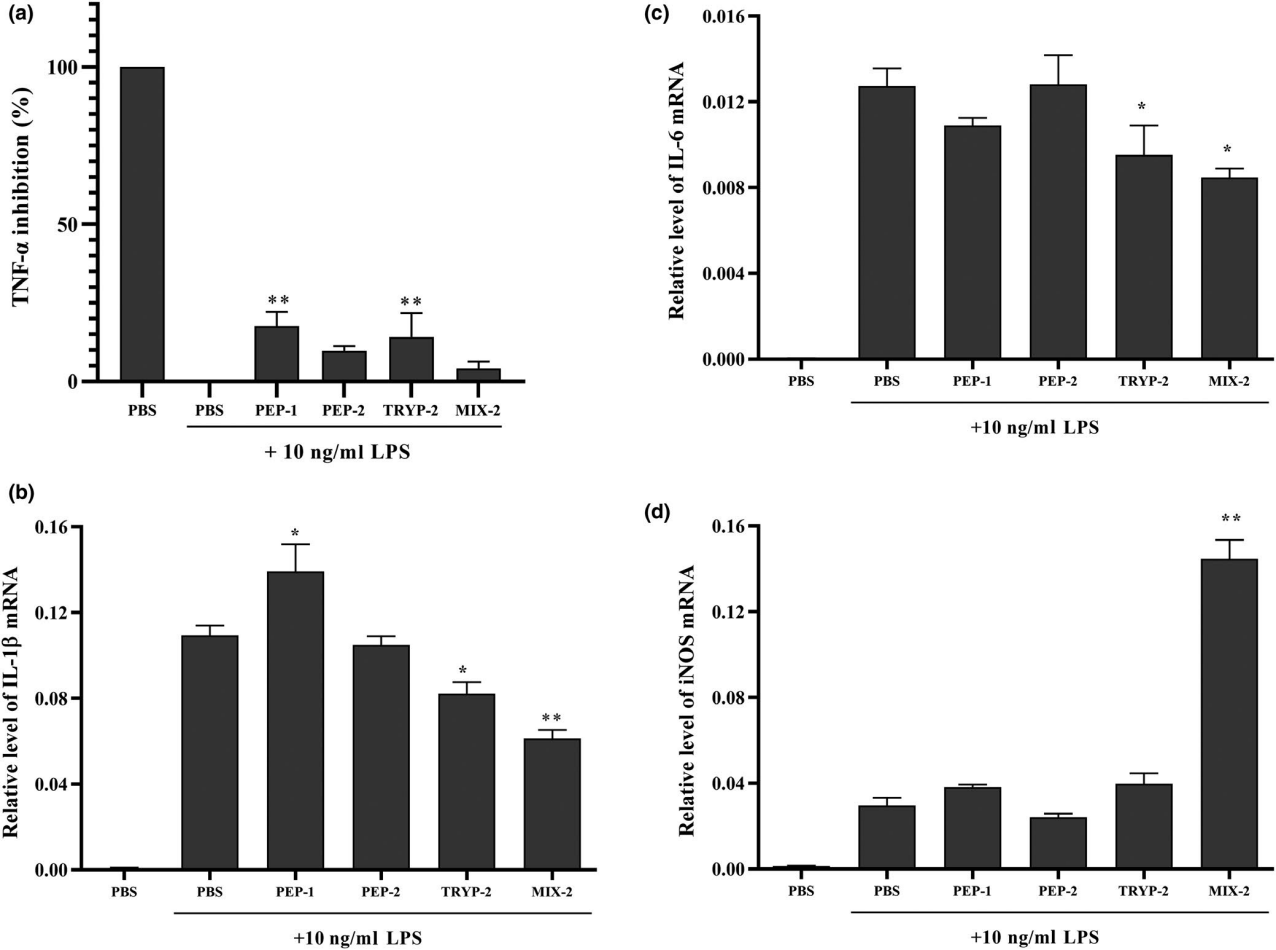
Effects of the bioactive peptide fractions on mRNA expressions of pro‐inflammatory mediators in RAW 264.7 mouse macrophage cells. (a) TNF‐α; (b) IL‐1β; (c) IL‐6; (d) i‐NOS. The final concentrations of the bioactive peptide fractions were 0.6 mg/ml in the initial culture media. All tests were conducted in triplicate, and mean values are used. The vertical bars represent the standard deviation of each data point. Values marked by the same letter are not significantly different versus PBS + LPS (**p < .01; *p < .05)

To investigate the effects of the bioactive peptide fractions on pro‐inflammatory mediator production, including TNF‐α, IL‐1β, IL‐6, and i‐NOS, RAW264.7 macrophage cells were pretreated with PBS and the bioactive peptide fraction solution followed stimulation with LPS. As shown in Figure 4, the secretion of TNF‐α was significantly inhibited by 0.6 mg/ml PEP‐1 and TRYP‐2 but not PEP‐2 and MIX‐2 (*p* < .01) (Figure 4a). The mRNA levels of IL‐1β, IL‐6, and i‐NOS increased dramatically after LPS stimulation but not in the control (PBS treatment), whereas IL‐1β, IL‐6, and i‐NOS level in treatment with various peptide fractions was attenuated in varying degrees (Figure 4b‐d). MIX‐2 and TRYP‐2 but not PEP1 and PEP‐2 repressed IL‐1β and IL‐6 mRNA expression significantly (*p* < .05), and MIX‐2 exhibited the strongest inhibition effect. However, MIX‐2 significantly increased i‐NOS mRNA expression compared with other peptide fractions (*p* < .05), and PEP‐1 did the same to IL‐1β.

## 4 DISCUSSION

Currently, enzyme hydrolysis is the major process for production of the bioactive peptides from food proteins aside from microbial fermentation (Chai et al., 2017). Maxipro PSP used in this study is a prolyl endopeptidase to produce peptides containing C‐terminal proline residue, which is hydrophobic and contributes to its antioxidant ability and is tolerance to the enteropeptidase in vivo (FitzGerald & Meisel, 2000). Also, Maxipro PSP was also used to improve the flavors of protein hydrolysates, especially for those from marine organisms. Additionally, compared with the traditional separation method based on molecular weight (Asha et al., 2016; Miao et al., 2018), the hydrophobic components are more easily obtained by gradient ethanol separation applied in this study. These might be the reason why MIX‐2 peptide fraction possessed the best hydrophobic in this study.

Commonly, higher content of hydrophobic amino acids (Pro, Tyr, Val, Leu, Ile, Phe and Met) in the peptide means its superior antioxidant activity, which might be attributed to an easy interaction with lipid‐soluble free radicals and delay lipid peroxidation, resulting in the enhancement of the antioxidant activity of the peptides (Harnedy & FitzGerald, 2012; Li & Yu, 2015; Zhu, Chen, Tang, & Xiong, 2008; Zou, He, Li, Tang, & Xia, 2016). Antioxidant activities of the four peptides fractions in this study were better than that of the peptides fraction OPHpap‐1, OPHpap‐2, and OPHpap‐3 fractionated by ultrafiltration with molecular weight cutoffs from oyster protein hydrolysates (Asha et al., 2016). The difference might be attributed to the peptides fractionation methods used in this study, which was based on hydrophobicity. The better antioxidant activities of peptide fractions PEP‐1, PEP‐2, TRYP‐2, and MIX‐2 were attributed to their hydrophobicity. Additionally, the amino acid composition also plays an important role in the antioxidant property, such as branched‐chain amino acids (Val, Ile, and Leu), antioxidant amino acids (Tyr, Met, His, Lys, and Trp), and the negatively charged acidic amino acids (Glu and Asp) (Miao et al., 2018; Yang, Yang, Li, Li, & Jiang, 2011; Zou et al., 2016). The free amino acid Tau can also contribute to the antioxidant property (Marcinkiewicz & Kontny, 2014

). Our results also revealed that the content of these mentioned amino acids in TRYP‐2 fraction was the most abundant, which might contribute to its optimal antioxidant activity. Certainly, the amino acid arrangement and size of peptide fragment are another nonnegligible contributor to antioxidant activity (Zou et al., 2016). It is easy to understand that these factors were determined by the protein hydrolysis process, such as enzyme used and experimental conditions (Lee, Saravana, Cho, Haq, & Chun, 2018; Sun, Shen, & Luo, 2011). In this work, under the same elution condition (40% ethanol), three antioxidant assays showed the same antioxidant capacity trend, MIX‐2 < PEP‐2 < TRYP‐2, which indicated that the enzyme used was an important factor.

It is well known that the antioxidative activities of bioactive compounds are directly correlated with their reducing power (Zhang et al., 2012). So, the reducing power of antioxidant candidates may serve as an important indicator of their antioxidant activity (Kaur, Arora, & Singh, 2008). ORAC assay is based on hydrogen atom transfer, and DPPH radical scavenging activity and FRAP assay are redox reaction based on electron migration. Taken together, the results of three antioxidant activity assay here suggested the antioxidant properties of these four peptide fractions from oyster.

Our results showed that the bioactive peptide fractions not only had no cytotoxicity but also promoted cell proliferation. It was same to the report that salmon byproduct protein hydrolysate fraction 1 (SPHF1) showed no toxicity effects on Chang liver and RAW264.7 macrophage cells (Ahn, Je, & Cho, 2012).

It is well known that overproduction of oxidants without control in vivo could cause a development of chronic inflammation, which is a risk factor for most human diseases. During development of inflammation, macrophage cells release some pro‐inflammatory mediators, such as TNF‐α, IL‐1β, IL‐6, and COX‐2, to further activate macrophage and promote other inflammatory cytokines production, deteriorating the pathophysiological conditions (Bamdad, Bark, Kwon, Suh, & Sunwoo, 2017). In the inflammatory response, TNF‐α is a key mediator and regulates the expression of other cytokines. IL‐6 and IL‐1β could promote the differentiation of B cells into plasma cells and secreting immunoglobulins and B‐cell proliferation. In addition, IL‐6 has a chemotactic effect on mononuclear macrophages to aggravate the inflammatory response around the hematoma (Ertenli et al., 2001). Also, in the pathological condition, i‐NOS was induced to synthesize a large amount of NO, causing toxic effects of cells by inhibition of mitochondrial function and destruction of energy metabolism, and concomitantly aggravation of the inflammatory response (Nakazawa et al., 2017). Cytokines as an important inflammatory mediator affect the metabolism of NO, and both TNF and IL‐1 could induce i‐NOS production (Kingery et al., 2017). So, down‐regulation of these mediators is considered as a signal in anti‐inflammatory therapy (Kiewiet, Gros, van Neerven, Faas, & de Vos, 2015).

Recent study showed that amino acids play an important regulatory role in inflammatory responses. Relative to the normal controls, the ulcerative colitis patients exhibited the significantly lower level of some antioxidant amino acids (Glu, Gln, Met, His) (He et al., 2018). And, amino acids (Gln, Leu, Pro) could repress expression of inflammatory gene *TNF* through inhibiting the NF‐κB pathway (van Meijl, Popeijus, & Mensink, 2010). Moreover, Gln exhibited the selective anti‐inflammatory effect, which decreased inflammatory factors (IL‐6, IL‐1β, IFN‐γ), but not to TNF‐α and IL10 (Addabbo et al., 2013). Our results also suggested that the peptide fraction we obtained here exhibited selective regulation on inflammatory mediators, which might be attributed to the amino acids compositions (Table 1). The same phenomenon was also demonstrated in several recent reports. For example, Inkanuwat et al. (2019) revealed that the anti‐inflammatory peptide SNPSVAGVR at 120 mM significantly upregulated i‐NOS and TNF‐α expression but downregulated IL‐6 expression. Ivan Chan‐Zapata, Arana‐Argáez, Torres‐Romero, and Segura‐Campos (2019) found that 10 μg/ml of the peptide fractions (1–3 kDa) isolated from *Salvia hispanica* L. seeds protein hydrolysates significantly induced an increase of NO level and a decrease of IL‐6, IL‐1β, and TNF‐α levels at the same time. It was not just limited to peptides, bioactive chemical Artepillin C also exhibited suppression effect on IL‐1β and TNF‐α, but no influence on IL‐6 and anti‐inflammatory cytokine IL‐10 (Szliszka, Mertas, Czuba, & Krol, 2013). However, further works need to focus on the antioxidant and anti‐inflammatory activity of these four peptide fractions in vivo.

## 5 CONCLUSION

In this study, four hydrophobic peptide fractions named as PEP‐1, PEP‐2, TRYP‐2, and MIX‐2 were obtained from enzyme hydrolysates of oyster soft tissue by pepsin, trypsin, and Maxipro PSP, respectively. All peptide fractions showed no cytotoxicity, and the fraction TRYP‐2 exhibited the best antioxidant capacity based on DPPH· scavenging capacity, FRAP and ORAC assay in vitro. In addition, the four peptide fractions also showed anti‐inflammatory activities, although to a different extent, by downregulated secretion of TNF‐α and mRNA expressions of pro‐inflammatory mediators IL‐1β and IL‐6 and i‐NOS in LPS‐stimulated RAW264.7 cells. These results suggest that the peptide fraction TRYP‐2 from oyster soft tissue hydrolysates might be a potential resource for natural anti‐inflammatory components.

## CONFLICT OF INTEREST

The authors declare no conflict of interest.
